# School-Based Mental Health Education: Program Effectiveness and Trends in Help-Seeking

**DOI:** 10.3390/ijerph22040523

**Published:** 2025-03-29

**Authors:** Jean Kirnan, Gianna Fotinos, Kelsey Pitt, Gavin Lloyd

**Affiliations:** Psychology Department, School of Humanities & Social Sciences, The College of New Jersey, Ewing Township, NJ 08618, USApittk1@tcnj.edu (K.P.); lloydg1@tcnj.edu (G.L.)

**Keywords:** youth, mental health, help-seeking, COVID, school, education

## Abstract

One of the strategies to address the persistent youth mental health crisis is school-based educational programming. This paper reports on two distinct studies regarding *Coming Up for AIR*, a school-based mental health education program: (1) program effectiveness, measured as gains in student mental health literacy; and (2) trends in help-seeking behavior before, during, and after the pandemic. A survey on program content was administered to assess program effectiveness. Data collected between 2020 and 2023 from four schools yielded 473 responses. A comparison of pre- and post-scores demonstrated statistically significant gains in program content. Mental health literacy improved across gender and grade level (8th, 9th, and 10th), as well as for students with prior exposure to a mental health curriculum. In the second study, help-seeking behavior was evaluated before, during, and after the pandemic. While other programs measure intention, *Coming Up for AIR* measures actual behavior as students can ask for help for themselves or a friend. Data did not reflect individual student responses, but rather were aggregated and provided the number of students per presentation who requested help. Archived declaration card data from January 2019 through February 2024 was accessed, representing 28 different schools and 16,289 middle and high school student responses. School-level data were analyzed by grade level (middle school or high school) and date (pre-, intra-, or post-COVID-19). Significant differences in self-referral were found for both grade level and presentation date. Self-referrals were significantly higher post-COVID-19 compared to pre-COVID-19 with middle schoolers increasing 90% and high school students increasing 36%. Analysis of friend referrals showed a significant difference for grade level, but not presentation date. Again, middle school students were more likely to make a referral than high schoolers. The data suggest that the mental health crisis in middle school students persists at an alarming rate. Schools are at the forefront of addressing mental health issues for youth. External educational programming can bring awareness to mental health concerns and promote help-seeking in youth.

## 1. Introduction

The prevalence of mental illness in grades K-12 is a pressing topic in schools, generating increased concern from educators and the community. The frequency of mental health disorders and suicide has prompted a variety of initiatives including educational programming and counseling resources. The COVID-19 pandemic with its resulting closures and restrictions added to this already dire situation. The purpose of this paper is twofold: (1) demonstrate the effectiveness of a youth mental health education program, *Coming Up for AIR*; and (2) investigate trends in program-initiated help-seeking behavior before, during, and after the pandemic.

### 1.1. Youth Mental Health Crisis

The Surgeon General of the United States (OSG, 2021) notes that even before the COVID-19 pandemic, both the percent of and persistent increase in youth with mental health concerns were alarming [1]. As of 2018–2019, 1 in 7 children aged 3–17 (13%) in the United States had a diagnosed mental or behavioral health condition. A 2021–2022 study of this same age group noted high diagnosis rates for anxiety (10%), behavioral disorders (7%), and depression (4%). In older youth, 12–17 years, mental health disorders are even more prevalent. Relying on 2021–2022 data reported by youth themselves, 21% identified with symptoms of anxiety and 17% with depression [2].

The social isolation, fear, and restricted access to health services, as well as food and housing insecurity brought about by COVID-19 contributed to the increase in mental health concerns for youth. In their review of 53 longitudinal studies across 12 countries tracking youth mental health concerns, Madigan et al. (2023) identified significant increases in depression and slight increases in anxiety among youth when contrasting pre- and post-pandemic [3]. The concerning numbers reported in the United States reflected a global crisis.

The most recent data from the Centers for Disease Control report a small improvement in feelings of sadness or hopelessness in high school students post-COVID-19. However, the reduction from 42% in 2021 to 40% in 2023 for this measure is still substantially higher than the 30% that was reported in 2013, pre-COVID. Additionally, other key indicators in the survey of considering suicide, making a plan, or attempting suicide did not show a meaningful change from the 2021 data. Alarmingly, 20% considered attempting suicide, 16% had developed a plan for suicide, and 9% attempted suicide [4].

Thus, the youth mental health crisis that had been increasing, accelerated with the pandemic. However, even with a return to more normal schooling and social interaction, most numbers remain elevated. An additional concern is raised when considering that only half (53%) receive treatment [2]. Further, 50% of all lifetime mental illness will manifest before the age of 14 [5]. Neufeld et al. determined that adolescents with a mental health disorder who did not receive treatment were seven times as likely to have a recurrence by the age of 17 [6]. Early diagnosis and treatment are critical as untreated conditions additionally impact youth educational outcomes and adult achievements. Children with mental health disorders have higher rates of delinquency and special education classification as well as being more likely to have lower test scores, repeat a grade, and drop-out of school. In adulthood, they achieve lower levels of education and earnings [7]. These findings highlight the need for and benefits of early intervention.

A key strategy for early intervention of mental health disorders lies in educational programming. Barriers for youth in seeking help for mental health disorders include low mental health literacy, negative attitudes (i.e., stigma), distrust, and negative parental attitudes as well as issues related to access to mental health resources [8]. Aimed at the barriers that restrict help-seeking, schools provide a variety of programs to improve mental health literacy by correcting misinformation and reducing stigma.

### 1.2. Mental Health Education Programs

Patafio et al. provide a broad review of mental health education programming [9]. Their review of articles detailing 126 interventions found that most programming occurred in schools (84%) and was universal (88%) in nature rather than targeting a specific youth demographic. The most commonly measured outcomes were mental health knowledge and attitudes (both appeared in over 70% of the studies) with help-seeking evaluated to a lesser extent (33% investigating some type of help-seeking—intention, behavior, or help for others). School-based interventions were the most successful in achieving the desired outcomes compared with programs delivered in the community or accessed via the internet. Of the 106 studies delivered in a school setting, improvements were noted most in mental health knowledge (86%), followed by attitudes (63%). In a narrower review of only school-based interventions, Ma et al. found moderate support for the effectiveness of programs in 22 studies [10]. Approximately 40% of the studies reported statistically significant outcomes for measures of knowledge (6 of 15) and attitudes (6 of 16). However, all three studies that reported a combined outcome measurement of knowledge and attitudes found statistically significant gains following program interventions. A similar combined measure of knowledge and attitudes is used in the current study and will be referred to as “mental health literacy”.

The review by Patafio et al. reported moderate success for help-seeking intentions (61%) and low success in improving help-seeking behaviors (29%). Studies of help-seeking for others were quite successful (86%) but the authors did not specify if these were intentions or behaviors. While help-seeking behavior has the lowest success at 29%, only a few studies measured this. Most of the 126 studies evaluated intention when considering help-seeking (31%), with only 8, or 6%, measuring behavior [9].

Salerno’s review of universal, school-based mental health awareness programs in the U.S., reported on 15 studies of programming in grades 5 through 12 [11]. Similar to Patafio et al.’s review, studies were more likely to measure knowledge and attitudes than help-seeking. Additionally, in agreement with other reviews, when a study did measure help-seeking, it was less likely to yield a successful outcome relative to gains in knowledge or attitudes. Salerno reported statistically significant gains as follows: 9 of 12 measuring knowledge, 8 of 11 measuring attitudes, and 4 of 7 measuring help-seeking. However, in this review, only 1 of 4 help-seeking outcomes appeared to measure behaviors–others measured attitudes toward help-seeking.

While the published research reveals some differences in the percent of programs reporting improvements in mental health literacy, the majority do show benefits. Similar to these educational programs, it is hypothesized that mental health literacy will show improvement following the *Coming Up for AIR* presentation.

**H_1_.**  *Post-scores of mental health literacy will be higher than pre-scores*.

### 1.3. Group Differences

Prior research suggests that both age and gender are associated with mental health literacy. While not studying a specific mental health program, Chandra and Minkowitz investigated mental health knowledge, attitudes, and willingness to use mental health services in a sample of 8th graders [12]. They found that girls had higher mental health knowledge and lower attitude scores than boys. In their evaluation of a school-based program, *Mental Illness Education*, Rickwood et al. investigated gender differences in the high school participants [13]. These researchers found that girls had higher pre-scores relative to boys but reported no interaction between gender and program. In other words, despite initial gender differences in knowledge and attitudes, boys and girls gained equally from pre- to post-measures. Campos et al. studied a school-based program, *Find Space for Mental Health* [14]. In a younger group of 12–14 year-olds, evidence was found for program effectiveness on a combined measure of knowledge and stereotypes with no difference in gains by gender. These findings are further supported by Wahl et al.’s investigation of *Breaking the Silence*, with middle school students and *Ending the Silence* with high school students which found no differences in program impact across gender [15,16]. Thus, girls may be more likely to score higher in pre-measures of mental health literacy, but do not differ in gains following the program presentation.

An early review of the literature by Wahl concluded that knowledge and understanding of mental illness improved when children grow older [17], as younger children lack information as to the specific symptoms of mental illness, causes, or treatment. However, empirical support for age differences has been mixed. Clark et al. investigated the relationship between mental health literacy and traditional masculinity norms in a sample of 12–18 year-old boys and reported lower mental health literacy in younger students [18]. Campos et al. did not report grade differences in the pre-measure, but did note variability in grade level gains with 9th grade students improving more than 7th graders following the *Find Space for Mental Health* intervention.

Consistent with prior research it is expected that boys will have lower scores on pre-measures of mental health literacy compared with girls. This difference will also hold for younger grades compared with upper grades. While the data suggesting no interaction is strong for gender, there are mixed findings for grade level differences in program effectiveness. Thus, despite grade level differences reported by Campos et al. [14], we proposed similar hypotheses of no interaction for gender or grade.

**H_2_.**  *Males will have lower pre-scores than females*.

**H_3_.**  *The rate of improvement from pre-scores to post-scores will not differ by gender*.

**H_4_.**  *Lower grades will have lower pre-scores than upper grades*.

**H_5_.**  *The rate of improvement from pre-scores to post-scores will not differ by grade*.

Wei et al. criticized earlier studies in that they often failed to measure and control for confounding factors such as age, gender, race, socio-economic status, exposure to similar mental health education programs, and mental health status of self and relevant others [19]. The *Coming Up for AIR* data provides information on gender, grade, ethnicity, and prior exposure to mental health programming. Small sample sizes preclude an analysis of the current data by ethnicity, but gender, grade level, and prior exposure were addressed. To our knowledge this is the first study to investigate the impact of prior exposure on current program effectiveness. While it is assumed that those with prior exposure will have greater mental health literacy at pre-measure, it is unclear if they will continue to benefit from additional programming. A strong argument can be made that a ceiling effect may occur and with less room for improvement and less novelty in the information presented, an interaction may be evidenced in that those with prior exposure will demonstrate weaker gains.

**H_6_.**  *Students with prior exposure to mental health education will have higher pre-scores than those without*.

**H_7_.**  *The rate of improvement from pre-scores to post-scores will be weaker in those with prior exposure to mental health education*.

### 1.4. Help-Seeking

Similar to Patafio [9], other reviews have found that help-seeking is less often measured than knowledge or attitudes and when measured, is usually defined by intentions rather than behaviors. Rickwood et al., like others, defined help-seeking as intention rather than behavior [13]. In their study of a school-based program, help-seeking showed a weak improvement compared with strong changes in knowledge and attitudes. After the program, students increased their willingness to seek help from a professional or a friend but showed no change when help-seeking was defined as a family member or school resource.

Wei et al. reviewed 27 studies and identified only 8 that investigated help-seeking [19]. Of these 8, only 3 measured behavior, and these were self-reports with mixed results. One study found no improvement, while the other two found improvement in help-seeking from some sources but not others. The other 5 defined help-seeking as intention or attitude.

Even when behavior, rather than intention, is reported, help-seeking is usually assessed via self-report. Youth often respond to questions of whether they have sought out or met with a mental health professional. In the *Coming Up for AIR* program, the measure of help-seeking is actual behavior in the form of a declaration card asking for help for oneself or a friend at the time of the presentation. Uniquely, the program provides an immediate means to seek help through the use of the cards and the presence of counselors.

There has been a notable increase in youth mental health disorders in recent years. However, a key question remains as to whether youth have increased help-seeking to reflect the greater number experiencing mental illness. Stelzmann et al. conducted a scoping review of mental health and help-seeking during the pandemic [8]. Their review of 14 studies confirmed increases in mental health disorders for adolescents and emerging adults, but a corollary increase in help-seeking was not found. This suggests that help-seeking would not vary across pandemic time periods. However, since *Coming Up for AIR* provides an immediate process for seeking help, it could be argued that help-seeking would increase. Due to the discrepancy between program purpose and published findings, non-directional hypotheses are proposed for help-seeking.

**H_8_.**  *Help-seeking for self and friend will differ across pre-, intra-, and post-pandemic periods*.

**H_9_.**  *Help-seeking for self and friend will differ by grade*.

### 1.5. Current Study

The program under study, *Coming Up for AIR*, is comparable to the educational programming reported in the literature as it is school-based and universal, with content focused on general information to improve mental health literacy and facilitate help-seeking. Similar to other programs reported in the literature, effectiveness was assessed by a comparison of pre- and post-measures. The data for mental health literacy allowed for an analysis of gender and grade level, as well as prior exposure, which has not previously been reported in the literature. Additionally, unique to this report is the measurement of help-seeking as an actual behavior, observed during the presentation, rather than self-reported at a later date. Thus, this study provides novel information on a program not previously reported in the published literature, an investigation of prior exposure, and a separate analysis of help-seeking defined as behavior and investigated across the pandemic years.

The two different data sets used to assess program effectiveness and help-seeking were obtained from Attitudes in Reverse, AIR, the non-profit organization that created and administers the *Coming Up for AIR* program. The research was approved by the Institutional Review Board at The College of New Jersey (protocol code 2019-0254; date of approval: 3 September 2022). Both studies followed a non-experimental design.

## 2. Materials and Methods

### 2.1. Study 1 Methods—Program Effectiveness: Mental Health Literacy

#### 2.1.1. Participants

Schools scheduled for the *Coming Up for AIR* presentation were invited to participate in a study to assess program effectiveness. All schools accepting the invitation were included resulting in data from four schools collected between 2020 and 2023. A total of 473 students in grades 8, 9, and 10 completed both pre- and post-surveys. There were slightly more females (54%) than males (44%), with approximately 2% indicating other for gender. The largest ethnicity reported was White at 56%, followed by Hispanic at 24%, with a sizable number indicating more than one race at 10%. Gender and ethnicity data are provided by grade level in Table 1.

#### 2.1.2. Materials

A mental health literacy survey reflecting the content of the *Coming Up for AIR* presentation was developed by the researchers. This strategy is similar to other researchers who developed assessments specifically for the program under study [13,15,16], *Mental Illness Education, MIE; Breaking the Silence; Ending the Silence*. This trend was also noted in several of the review articles and Mellor found that in the majority of studies (13 of 17), program effectiveness was measured by a survey developed to reflect the specific program content [19,20]. While some studies measured knowledge and attitudes separately, a single measure of mental health literacy was used here in a manner similar to other researchers [14,21,22,23]. The survey developed for *Coming Up for AIR* included 21 items that were answered on a 4-point Likert scale (1 = *strongly disagree* to 4 = *strongly agree*). To prevent an acquiescence response set, 12 items were reverse scored. The readability level was calculated using the Flesch-Kincaid test to ensure that the measures could be easily understood by middle school and high school students. The measures were found to be at a 6.5 grade reading level. Reliability was assessed as internal consistency and found to be acceptable (α = 0.83).

Several steps were taken in the development of the survey to ensure content validity. After attending presentations and examining the materials, two researchers independently identified major themes, developed survey items to reflect program content, and confirmed that *Coming Up for AIR* compared favorably to similar programs reported in the published literature. An initial pool of 39 items were reviewed by program developers at AIR. Following an iterative process, the number of items was reduced ensuring that the most critical program concepts were covered, while others were modified to improve scope, clarity, or readability.

Additional demographic items were included requesting information on gender, ethnicity, grade level, and prior exposure to mental health education courses or programming. Pre- and post-measures both included the mental health literacy survey and demographics. The post-measure also contained four items measuring reactions to the program.

#### 2.1.3. Procedure

The *Coming Up for AIR* program, copyrighted by AIR, is a 60-min presentation combining contact and education-based methods for intervention. Students engage with a PowerPoint, a video, and an interactive discussion. Additionally, participants hear a personal story about a struggle with mental illness. During each presentation, students are given an opportunity to fill out declaration cards to ask for help for themselves or for a friend. School counselors are present during the presentation and then follow up with each student who is identified through the declaration cards. Program presenters are trained by AIR. A unique aspect of AIR’s educational programs is that there is always at least one certified therapy dog in attendance for students to interact with after a presentation. Additionally, all dogs and dog handlers are trained and screened for work in school settings. As of 2024, over 250,000 students in the United States have participated in the *Coming Up for AIR* program [24].

The schools administered the pre-survey within a week before the presentation and the post-survey within a week following the presentation. Students recorded unique identification numbers, which were used to match the pre- and post-surveys. Student identification numbers could not be traced to student names by the research team. Both pre- and post-measures were administered online. The research was approved by the Institutional Review Board at The College of New Jersey (protocol code 2019-0254; date of approval: 3 September 2022).

### 2.2. Study 2 Methods—Help-Seeking Behavior

#### 2.2.1. Participants

Declaration cards were collected after every presentation given between January of 2019 and February 2024. Data represented 63 presentations at 28 different schools and included 16,289 middle and high school student responses. Out of the sample, 15 were presentations to middle school students while the other 48 were to high schoolers. Data collection spanned pre-, intra- and post-COVID-19 dates with 21, 26, and 16 presentations, respectively. Declaration information was aggregated after each presentation and recorded as the number of students requesting help for self or for a friend. Unlike Study 1 which reported on individual student data, this archived data was at the school/grade level and did not include demographic information such as gender or race.

#### 2.2.2. Materials

Help-seeking was measured with the declaration cards that were distributed during every presentation. Students have the opportunity to ask for help for themselves (self-referral) and/or for a friend (friend referral). The card information includes student names, but is collected in a confidential manner and reviewed by school counseling staff. While the cards themselves include names to ensure students are connected with help, researchers did not have access to the original cards (N = 16,289) with student names. Researchers only had access to school and grade-level summaries as described above.

#### 2.2.3. Procedure

The *Coming Up for AIR* presentation content was identical to Study 1. Towards the end of all presentations, students were invited to complete a declaration card, while at the same time school counselors were identified and would follow up with requesting students. The percent of cards returned were tallied separately for self and friend by the schools.

Grade level was operationally defined as middle school (grades 6, 7, and 8) and high school (grades 9, 10, 11, and 12). The date of presentation was defined as pre-COVID-19 (January 2019 to March 2020), intra-COVID-19 (October 2020 to May 2022), and post-COVID-19 (October 2022 to February 2024). The research was approved by the Institutional Review Board at The College of New Jersey (protocol code 2019-0254; date of approval: 3 September 2022).

## 3. Results

### 3.1. Study 1 Results—Program Effectiveness: Mental Health Literacy

A series of repeated measures analyses of variance (ANOVA) were conducted to determine program effectiveness overall as well as main effects of gender, grade level, and prior exposure. Pre-and post-mental health literacy scores are reported in Table 2 along with ANOVA results for the main effects and interactions. Sample sizes vary slightly for gender and grade level due to missing data but are notably less for prior exposure. The prior exposure item required participants to select all that apply from a list indicating possible sources of mental health education, such as teacher covered in class or attended a different mental health program. The current study only analyzed those who indicated they had no previous exposure to mental health education (249) and those who indicated they had exposure from a teacher covering these topics in class (131). Response options with a low endorsement rate as well as individuals selecting multiple options were not included in this analysis.

**Figure 1:**
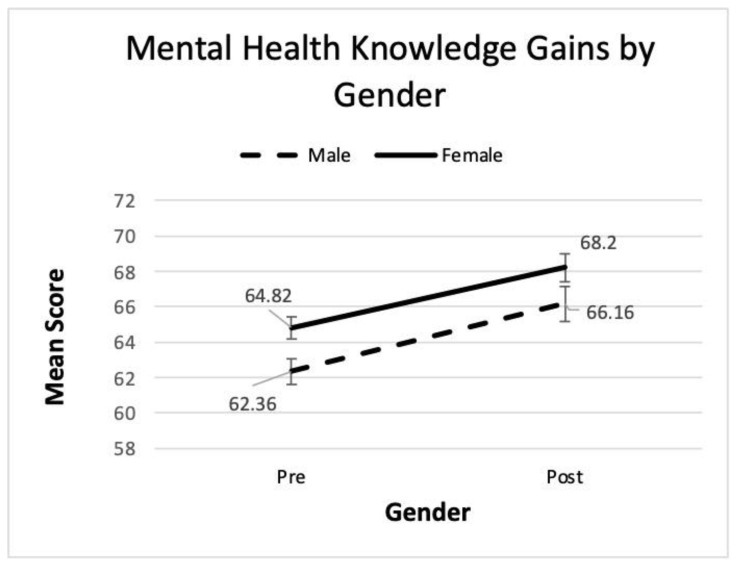
Line graph of pre- and post- mental health literacy scores by gender.

**Figure 2:**
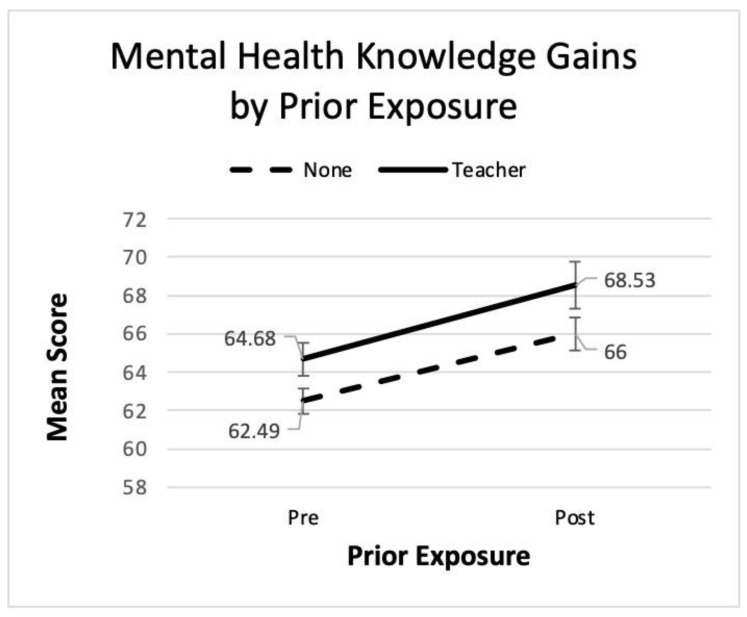
Line graph of pre- and post- mental health literacy scores by prior exposure.

The repeated measures ANOVA identified a significant main effect for time in all three analyses. Significant main effects were also found for gender, grade level, and prior exposure, with females, older students, and those with prior exposure scoring higher. Partial eta squared indicates a large effect size for time, but a small effect for the other main effects despite their statistical significance. The interaction terms for gender and prior exposure were not significant indicating that regardless of differences in mental health literacy prior to the *Coming Up for AIR* program, boys and girls, as well as those with and without prior exposure, all showed similar gains. See Figure 1 and Figure 2 where the lines for gender and prior exposure appear parallel, further enforcing the finding of no interaction.

**Figure 3:**
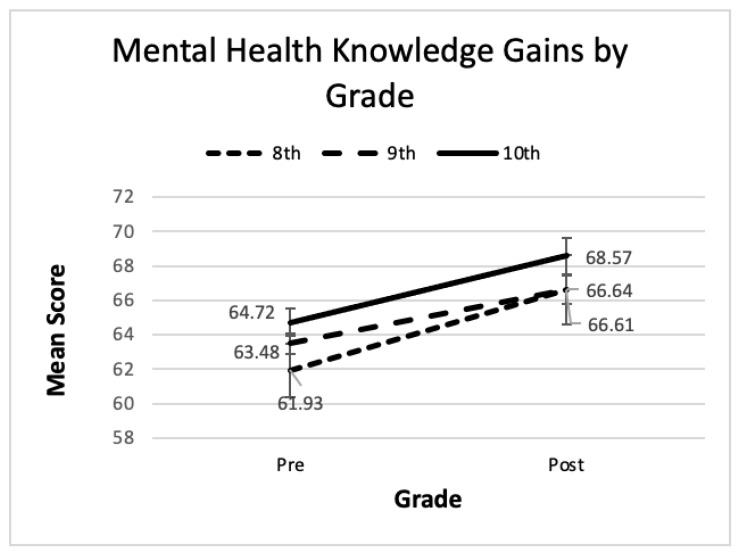
Line graph of pre- and post- mental health literacy scores by grade.

The interaction term for grade was marginally significant. While a visual inspection of Figure 1 and Figure 2 reveals parallel lines for the gender and exposure groups, in Figure 3, not all lines are parallel. The lines for 8th and 10th are roughly parallel, but 9th grade appears flatter suggesting a slower rate of improvement. Tukey’s post hoc pairwise comparisons revealed that 10th graders scored higher than 8th or 9th when averaging across the two time periods, but there was no difference between 8th and 9th grades. Thus, all grades improved from pre- to post-session, but 10th grade improved at a faster rate. Refer to Figure 3 for a visual representation of these results.

### 3.2. Study 2 Results—Help-Seeking Behavior

**Figure 4:**
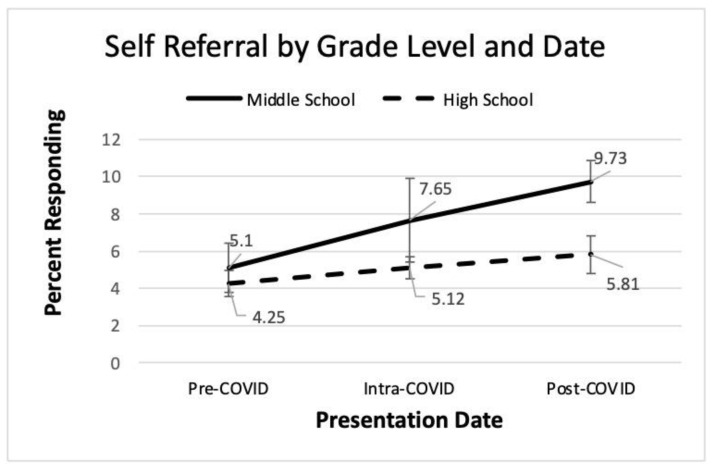
Line graph of self-referral by grade and date.

A 3 × 2, COVID-19 date by grade level, analysis of variance of help-seeking for oneself was conducted. Results for this analysis appear in Table 3. The percentage of students seeking help for self was statistically significant for both the main effects of COVID-19 date and grade level, with partial eta squared indicating a moderate effect size. Middle school students were more likely to seek help for themselves than their high school counterparts. A Tukey post hoc analysis of COVID-19 date revealed statistically significant differences between pre- and post-COVID-19 dates, with post-COVID-19 reporting a greater percent of students seeking help. The interaction term was not significant. These findings appear visually in Figure 4 where a continual increase in help-seeking for self is evident over the three time periods for both grade levels.

**Figure 5:**
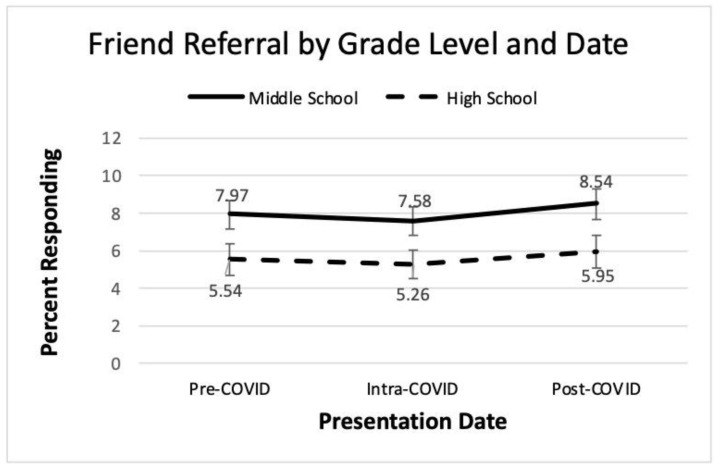
Line graph of friend referral by grade and date.

A 3 × 2, COVID-19 date by grade level, analysis of variance of help-seeking for a friend was conducted. These results also appear in Table 3. The percent of students seeking help for a friend was statistically significant only for the main effect of grade level, with partial eta squared indicating a moderate effect size. Middle school students were more likely to seek help for a friend than high school students. Neither the main effect of COVID-19 date nor the interaction term were significant. These findings appear visually in Figure 5 where there is a slight dip in help-seeking during COVID-19 and a small uptick post-COVID-19 for both grade levels.

## 4. Discussion

This study provides support for the effectiveness of the *Coming Up for AIR* program. A comparison of pre- and post- mental health literacy scores revealed improvement across gender, grade level, and prior exposure. Additionally, a second dataset revealed patterns in help-seeking behavior before, during, and after COVID-19.

Gains in mental health literacy affirm Hypothesis 1 and offer agreement with earlier research that demonstrated increases in mental health literacy following other mental health program presentations [9,10,11]. However, because this study was a non-experimental design lacking a control group, alternative explanations for the gain in scores must be considered. It is possible that information on mental health was gained from an external event such as a mental health initiative in the school or community or communications from the school counseling staff. Additionally, the study is open to the influence of testing in that the pre-survey itself may have sparked an interest in mental health leading students to seek information or engage in conversation about the topic. While the short time period between the pre- and post-test makes these influences less likely, they are still uncontrolled factors.

Hypotheses 2 and 3 were similarly supported in that this study reported lower pre-scores for boys but no difference in gains for boys and girls, providing agreement with earlier research [12,13,14,15,16]. Historically, lower scores for boys have been associated in the literature with perceived stigma surrounding masculine ideals and perceived parental disapproval [12].

Hypothesis 4 was supported in that lower grades scored worse in the pre-measure, agreeing with earlier research [17,18]. However, Hypothesis 5 was partially supported. While this study proposed no differences in gains for lower or upper grade levels, the interaction term was marginally significant, technically supporting Hypothesis 5. However, a post hoc analysis suggested a significantly stronger impact of the program for 10th graders. Prior research by Campos et al., found that 9th graders scored higher than 7th graders [18]. The disagreement in the current study between the interaction term significance level and the post hoc comparisons suggests statistical issues with discrepancies in sample sizes and makes it difficult to draw firm conclusions regarding grade level or age. The sample for 8th grade (N = 54) was substantially less than that for 9th (N = 244) or 10th (N = 177). A larger sample for each grade and extending the number of grade levels under study to include lower grades such as 6th and 7th and higher grades such as 10th, 11th and 12th would shed light on the influence of age or grade. At this time, we must conclude that any observed grade differences are likely due to sample variability and true differences would need confirmation in large and more robust samples.

Researchers need to balance statistical significance with practical significance and thus consideration should be given to the effect sizes of statistically significant findings. Partial eta squared was large for mental health literacy differences between pre- and post-test, suggesting meaningful change. However, effect sizes were small for gender, age, and prior exposure, despite these factors being statistically significant, raising questions as to the practicality of these sub-group differences.

The current research is one of the first to demonstrate pre-score differences for those with prior exposure supporting Hypothesis 6. However, Hypothesis 7 was not supported. Unexpectedly, and contrary to Hypothesis 7, there was no significant interaction for prior exposure; students with prior exposure gained similarly to those without. This suggests that mental health educational programming may be beneficial to students who are already receiving this information in the school curriculum. One might postulate that repeated messaging, messaging from multiple sources, and messaging at different points in time are crucial in enhancing mental health literacy in youth.

The current study measured help-seeking over time in an effort to investigate differences in grade level as well as possible trends pre-, intra-, and post-COVID-19. Hypothesis 8 proposed that help-seeking would differ over the COVID-19 dates. Figure 4 shows a gradual increase in help-seeking for self over time, but only the comparison of pre- and post-COVID-19 was statistically significant. Figure 5 shows a slight dip and then an increase in help-seeking for a friend over time, but these differences did not achieve statistical significance. These findings provide some agreement with research by Stelzmann et al. that failed to find increases in youth help-seeking during COVID-19 [8]. However, in regards to Hypothesis 9, strong grade level differences were noted. Middle school students were more likely to seek help for both themselves and friends compared to high schoolers.

### 4.1. Program and Study Strengths

The current study confirms commonly reported findings for general program effectiveness, effectiveness across gender and grades, and differences in pre-scores with boys and lower grades scoring worse than girls and higher grades. This study is the first to report such findings on the specific program, *Coming Up for AIR*. This research is also the first to examine mental health education program effectiveness for students with prior exposure to such information from a teacher. Interestingly, regardless of differences in pre-scores all groups reported similar gains after the program.

A major strength of the AIR program is the ability to measure observable help-seeking behaviors rather than behavioral intentions or self-reported behavior which are more dominant in the published literature [9,13,19]. Through the use of the declaration cards, AIR provides a means by which students can ask for help immediately. The collaboration of AIR with school counselors is essential for the success of this aspect of the program as counselors intercede by following up with each student, assessing the severity of their mental health concerns, and identifying internal and/or external resources for support.

The published literature suggests that some of the reluctance to seek help is tied to issues of access such as lack of knowledge regarding formal services [8] and the need for information on how to help a friend access mental health resources [13]. The *Coming Up for AIR* program provides immediate access to school mental health resources. This may explain the high levels of help-seeking in this study and observed by counselors.

### 4.2. Limitations and Future Directions

It should be noted that many of the review articles identified substantial and consistent methodological weaknesses in the published literature on mental health educational programming. They noted that studies often lacked a control group and that when a control group was included there was a lack of random assignment of participants to experimental and control conditions [9,11,19]. Wei et al. [19] called for the inclusion of confounding variables and noted that more rigorous methodology, including randomized control trials would help reduce the impact of unaccounted variables. Additional concerns include lack of standardized measures and inconsistencies in the use and timing of pre, post, and follow-up measures. These problems were apparent in the current study. Reported findings are limited by the lack of a control group and the use of non-standardized measures. In fairness to researchers, it is extremely difficult to gain access to students, especially post-COVID-19. Schools continue to be overwhelmed by deficits in academic achievement and increased student needs. While researchers should still strive for more rigorous methodologies, random assignment and follow-up measures may be more difficult to obtain.

As noted earlier, when measuring program effectiveness as gains in mental health literacy, a larger sample would have allowed for more detailed investigation of grade level. A larger and more diverse sample would also allow for an analysis of ethnicity to determine if the program is equally effective across various ethnic groups. Additionally, the confounding factors that Wei et al. [19] identified (i.e., socioeconomic status, mental health of the respondent, mental health of significant others in the respondent’s life, mental health crisis at the school) could be investigated in a larger and more diverse sample.

Similarly, help-seeking would benefit from a larger sample. While the data represent over 16,000 student responses, the data were provided and analyzed at the school/grade level. Thus, the sample size for statistical analysis was small at 63 presentations. A visual inspection of Figure 4 and Figure 5 suggests trends and group differences, but these did not achieve statistical significance, which may be related to the small sample.

Studies can also be improved by measuring outcomes at multiple points in time—immediately after an educational program and then at one or more later time periods. This would add to an understanding of the long term impact of mental health educational programming. It is possible that gains are temporary, and scores will drop over time. However, it is also possible that the impact of a program is not felt immediately. Anecdotal data suggest that youth do recall the information received and have accessed relevant resources when needed, albeit at a later date.

The data available was not conducive to an analysis of whether the *Coming Up for AIR* program increased help-seeking, as every presentation included the declaration card, and the purpose of the current study was to analyze trends in help-seeking over time. Thus, help-seeking in a control group that did not attend the presentation was impossible to obtain. This, of course, leaves any changes in help-seeking open to alternative explanations. There has been an increased awareness of mental health issues in general and increased resources in schools, particularly post-COVID-19. These may have influenced help-seeking by youth. However, anecdotal feedback from school counselors suggests that the program does make a difference as they report more youth seeking help after the presentation. Twelve counselors responded to a short, optional survey indicating program strengths and specific evidence of impact. Most observed that the strength of the program included the factual information, the impactful personal story, and the opportunity to interact with therapy dogs. When asked how they knew students were impacted, counselors described student conversations and behaviors after the presentation. Students mentioned the program to friends, were comfortable making referrals, and made comments on specific program information. One counselor stated: “…. the fact that they mentioned it to friends. Additionally, the fact that they referred friends. I think that says a lot about the program”. Another noted: “The presentation helps to reduce the stigma of asking for help. Our referral numbers reflect this”.

## 5. Conclusions

School-based mental health educational programming consistently demonstrates gains in mental health literacy from pre- to post-program measures. Gains are evident across various demographic factors such as gender, grade, and prior exposures. Gains appear to be equivalent despite differences in pre-scores across these demographics. Such findings suggest continued use of mental health educational programming for all students despite demonstrations of higher prior literacy levels. However, gains in mental health literacy are insufficient if youth do not ask for and receive help. The current study found that requests for help increased post-COVID-19 and are more prevalent in younger grades. Schools and educators are encouraged to provide effective mental health education programming, as well as means for youth to seek support. Such interventions are especially needed for middle school students who demonstrated greater help-seeking than their high school counterparts. The *Coming Up for AIR* program offers one such method.

Schools continue to provide a wide array of services and resources for their students, well beyond their primary mission of education. An already growing mental health crisis has been accelerated by the constraints of the COVID-19 pandemic. More youth are experiencing depression and anxiety, and efforts must be made to create awareness and promote help-seeking. Educational programming that destigmatizes mental health disorders, corrects misinformation, and promotes help-seeking can prove a valuable tool for educators.

## Figures and Tables

**Table 1:** Student Demographics by Grade.

	Grade			
Demographic	8th	9th	10th	Total
Gender				
Male	43.40%	45.87%	41.24%	43.86%
Female	52.83	53.31	55.90	54.24
Other	3.77	0.83	2.82	1.91
Ethnicity				
White	37.04	62.40	54.80	56.66
Hispanic	22.22	20.66	29.94	24.31
Black or African-American	20.37	3.31	3.39	5.29
Asian	1.85	2.48	1.13	1.90
Other	5.56	0.83	0.56	1.27
More than 1 Race	12.96	10.33	10.17	10.57
Sample Size	54	242	177	473

**Table 2:** Means, Standard Deviations, and Repeated-Measures ANOVA Statistics of Mental Health Literacy by Demographics.

Demographic	Pre		Post		ANOVA				
	*M*	*SD*	*M*	*SD*	Effect	*F*	*df*	*p*	η_p_^2^
Gender (N)									
Male (206)	62.36	5.29	66.16	7.14	Time	250.92	1, 461	<0.001	0.352
Female (256)	64.82	5.10	68.20	6.67	Gender	18.48	1, 461	<0.001	0.039
					T × Gender	0.87	1, 461	0.351	0.002
Grade (N)									
8th (54)	61.93	5.86	66.61	7.58	Time	208.47	2, 472	<0.001	0.306
9th (244)	63.48	4.94	66.64	6.56	Grade	5.59	2, 472	0.004	0.023
10th (177)	64.72	5.52	68.57	7.22	T × Grade	2.65	2, 472	0.071	0.011
Prior Exposure (N)									
No (249)	62.49	5.24	66.00	6.79	Time	203.05	1, 378	<0.001	0.349
Yes (131)	64.68	5.10	68.53	7.11	Prior Exposure	15.14	1, 378	<0.001	0.039
					T × PE	0.46	1, 378	0.500	0.001

Note. ANOVA = analysis of variance; T = time.

**Table 3:** Means, Standard Deviations, and ANOVA Statistics of Help-Seeking by Grade and Date.

	COVID-19 Date										
Help-Seeking/Grade	Pre		Intra		Post		ANOVA				
	*M*	*SD*	*M*	*SD*	*M*	*SD*	Effect	*F*	*df*	*p*	η_p_^2^
Self (N)											
Middle School (15)	5.10	2.59	7.65	4.53	9.73	2.19	Grade	8.24	1, 57	0.006	0.126
High School (48)	4.25	2.54	5.12	2.11	5.81	3.63	Date	4.36	2, 57	0.017	0.133
							Grade × Date	1.08	2, 57	0.347	0.030
Friend (N)											
Middle School (15)	7.97	2.45	7.58	2.61	8.54	2.68	Grade	8.32	1, 57	0.006	0.127
High School (48)	5.54	2.94	5.26	2.68	5.95	3.14	Date	0.30	2, 57	0.745	0.020
							Grade × Date	0.01	2, 57	0.992	<0.001

## Data Availability

Archived data were provided to the researchers by Attitudes in Reverse, AIR, the non-profit that developed and administered the *Coming Up for AIR* mental health education program. Data are not available as they are proprietary to AIR.
